# Metal artifacts reduction in kV-CT images with polymetallic dentures and complex metals based on MV-CBCT images in radiotherapy

**DOI:** 10.1038/s41598-023-35736-x

**Published:** 2023-06-02

**Authors:** Xiaochen Ni, Zhonghua Shi, Xinmao Song, Tianci Tang, Shengwei Li, Zhenfeng Hou, Wei Zhang, Wei Fang Wang, Fu Chen, Ji Li, Gang Yang, Ruichen Li, Xiaoshen Wang

**Affiliations:** 1 Department of Radiotherapy, Eye & ENT Hospital, Fudan University, 83 Fenyang Road, Shanghai, 200031 People’s Republic of China; 2 Shanghai United Imaging Healthcare Co., Ltd, Shanghai, 201800 People’s Republic of China; 3 Fudan University, Jiangyue Road 2600, Shanghai, People’s Republic of China

**Keywords:** Medical imaging, Physics

## Abstract

This paper proposes a metal artifact reduction method of using MV-CBCT images to correct metal artifacts in kV-CT images, especially for the complex metal artifacts caused by multi-metal interaction of patients with head and neck tumors. The different tissue regions are segmented in the MV-CBCT images to obtain template images and the metal region is segmented in the kV-CT images. Forward projection is performed to get sinogram of the template images, kV-CT images and metal region images. Artifact images can be reconstructed through those sonograms. Corrected images is generated by subtracting the artifact images from the original kV-CT images. After the first correction, the template images are generated again and brought into the previous step for iteration to get better correction result. CT data set of 7 patients are used in this study, compared with linear interpolation metal artifact (LIMAR) and normalized metal artifact reduction method, mean relative error of CT value is reduced by 50.5% and 63.3%, noise is reduced by 56.2% and 58.9%. The Identifiability Score of the tooth, upper/lower jaw, tongue, lips, masseter muscle and cavity in the corrected images by the proposed method have significantly improved (*P* < 0.05) than original images. The artifacts correction method proposed in this paper can effectively remove the metal artifacts in the images and greatly improve the CT value accuracy, especially in the case of multi-metal and complex metal implantation.

## Introduction

With the development of radiotherapy technology, radiotherapy has entered the stage of precise radiotherapy, in which the delineation of the tumor target area and organs and the calculation planning dose through kV-CT images of patients are the most critical basis for precise radiotherapy. However, when there are metal implants in the human body, almost all photons are absorbed when X-rays pass through the metal area because of high electron density of metal, and the number of photons are detected after penetrating the metal by the detector is not enough to reconstruct the complete image. This is photon starvation, which is also the main reason for metal artifacts. In addition, due to beam hardening, the hardened X-ray is easier to penetrate the soft tissue behind the metal, resulting in the deviation of the attenuation signal of the soft tissue. When the attenuation coefficient of different areas is discontinuous, it will cause the first derivative of the projection data to show weak continuity in a certain segment. After filtering, this weak continuity is further enlarged, and the light and dark strip artifacts are formed in the reconstructed images^1^, resulting in poor image quality and a serious impact on subsequent applications. The artifacts caused by metal in the area of head-and-neck are very serious, and it is difficult to accurately delineate the target area and organs^2^. To address this issue, various metal artifacts correction methods have been proposed.

The current metal artifacts correction methods mainly include: the projection images interpolation correction method^3–5^, iterative reconstruction method^6–9^, prior image correction method^10^, dual-energy/energy spectrum computed tomography correction method^11–13^ and correction method based on deep learning^14–18^. The projection images interpolation correction method uses the projection data of the surrounding normal tissue to perform linear interpolation on the metal area^3^, or normalizes the projection images by using the prior images and performs linear interpolation^4^, and obtain the correct images through back projection. Prior image correction method is using kV-CT to segment different issues in images to get prior images without metal artifacts and extract differences in sonograms to correct images. The iterative reconstruction method compares the calculated projection of the reconstructed images with the actual projection and uses the different images to correct the original reconstructed images. The deep learning-based correction method set up a training on a dataset consisting of images containing metal artifacts and images that do not contain metal artifacts, the convolutional neural network for metal artifacts correction realizes the correction of metal artifacts.

The X-rays energy of MV-CT or MV-CBCT is much higher than that of kV-CT, and the metal artifacts will be greatly weakened. Therefore, the dual-energy correction method is one of the most promising technologies to reduce metal artifacts^19–22^. MV-CT or MV-CBCT has been used in many studies to reduce metal artifacts, and has achieved a good effect in reducing artifacts for small or simple metals such as bone nails and steel plates^23–26^. Gao et al. proposed a method for the removal of metal artifacts from MV-CBCT and kV-CT images and improve the quality of kV-CT images in which artifacts are not severe^27^, and the CT value is not accurate in mouths because of using the CT value of MV-CBCT. Kim et al. combined deep learning and MV-CT images and proposed a new MAR method architecture^28^. First, the artifact-free kV-CT images were generated by MV-CT, and then the true kV-CT images are corrected by the second network combined with artifact-free kV-CT images. The method successfully suppresses metal artifacts, but needs a lot of data for training, and is not applicable to rare cases.

Although the above method eliminates metal artifacts to a certain extent and achieves a good correction result for a single small metal, however, for patients with head and neck tumors who have polymetallic dentures and complex metal^2^, such as a patient implanted multiple metal dentures, the area contaminated by metal artifacts is large and complex, the existing correction methods, such as the linear interpolation (LIMAR)^3^ and normalized interpolation (NMAR)^4^, are difficult to restore the true information of the image during the interpolation repair process, and the repair effect of the image is limited due to the serious artifacts^22,29^. In this paper, the MV-CBCT image is used to obtain more complete organizational structure information, and the iterative method is used to obtain accurate CT values and repair the details step by step, which can achieve good repair effect for metal artifacts in various situations.

## Materials and methods

### Equipment and patient selection

The image data used in this paper were obtained by scanning on the CT-accelerator-integrated uRT-linac 506c linear accelerator system produced by Shanghai United Imaging Healthcare Co., Ltd. The uRT-linac 506c accelerator contains an MV-CBCT imaging system and a diagnostic kV-CT imaging system. The original design concept of this type of accelerator integrates diagnostic CT and linear accelerator into one treatment room. The patient can complete the image scanning of MV-CBCT and kV-CT upon one setup in the same treatment room. In addition, the uRT-linac 506c is configured with FBCT and CBCT at the same time, which makes the registration of MV-CBCT and kV-CT not need to consider complicated situations such as patient positioning. The simulated head phantom used in this study is CIRS model 038 (CIRS, Norfolk, VA, USA). The enrolled patients were from September 2021 to February 2022 in the Radiotherapy Department of the Eye and ENT Hospital of Fudan University. Seven patients with nasopharyngeal carcinoma with multiple dentures were treated (using the eighth edition of AJCC staging).

**Figure 1:**
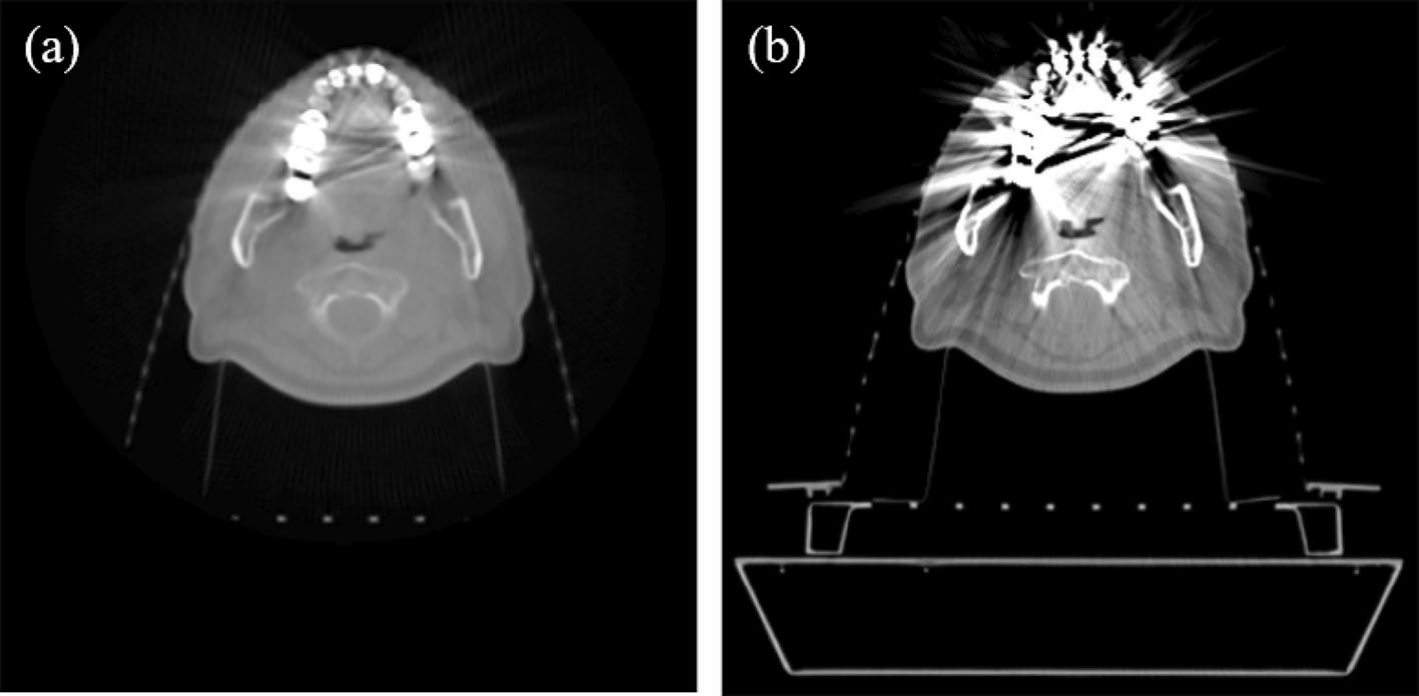
MV-CBCT images (**a**) and kV-CT images (**b**) of same slice of same patient.

The X-ray energy of the linear accelerator MV-CBCT imaging system is 1.5 MV. After the MV-CBCT images and the kV-CT images are obtained by scanning in the same body position (Fig. 1), the two sets of images are registered to the same frame of reference. The MV-CBCT image reconstruction matrix is 512 × 512, the pixel spacing is 0.5 mm × 0.5 mm, and the slice thickness is 0.5 mm, the kV-CT image reconstruction matrix size is same as MV-CBCT, the pixel spacing is 0.8 mm × 0.8 mm, and the slice thickness is 1 mm. The MV-CBCT images are segmented by the local threshold to obtain high-density area, medium-density area, and low-density area, and the average CT value of different areas in the kV-CT images is used to fill the segmented area to obtain template images. At the same time, the metal region is segmented in the kV-CT images. Forward projection is performed to get the sinogram of template images, kV-CT images, and metal region images. Then the difference images between the sinogram of the template images and the kV-CT images is calculated to extract the projection range of the metal region in the different images. Then the artifact images are obtained by back-projecting the projection range of the metal region, the corrected images can be generated by subtracting the artifact images from the original kV-CT images. After the first correction, the template images are generated again and brought into the previous step for iteration, after each iteration of generating the corrected image, the corrected kV-CT images are pre-projected and the template images are generated, the sonogram and template image are updated, and the details are repaired step by step. When reaching the preset number of iterations, the iteration times are normally 3 or 4, and the correction of the original kV-CT images is complete.

### Template images generation method

**Figure 2:**
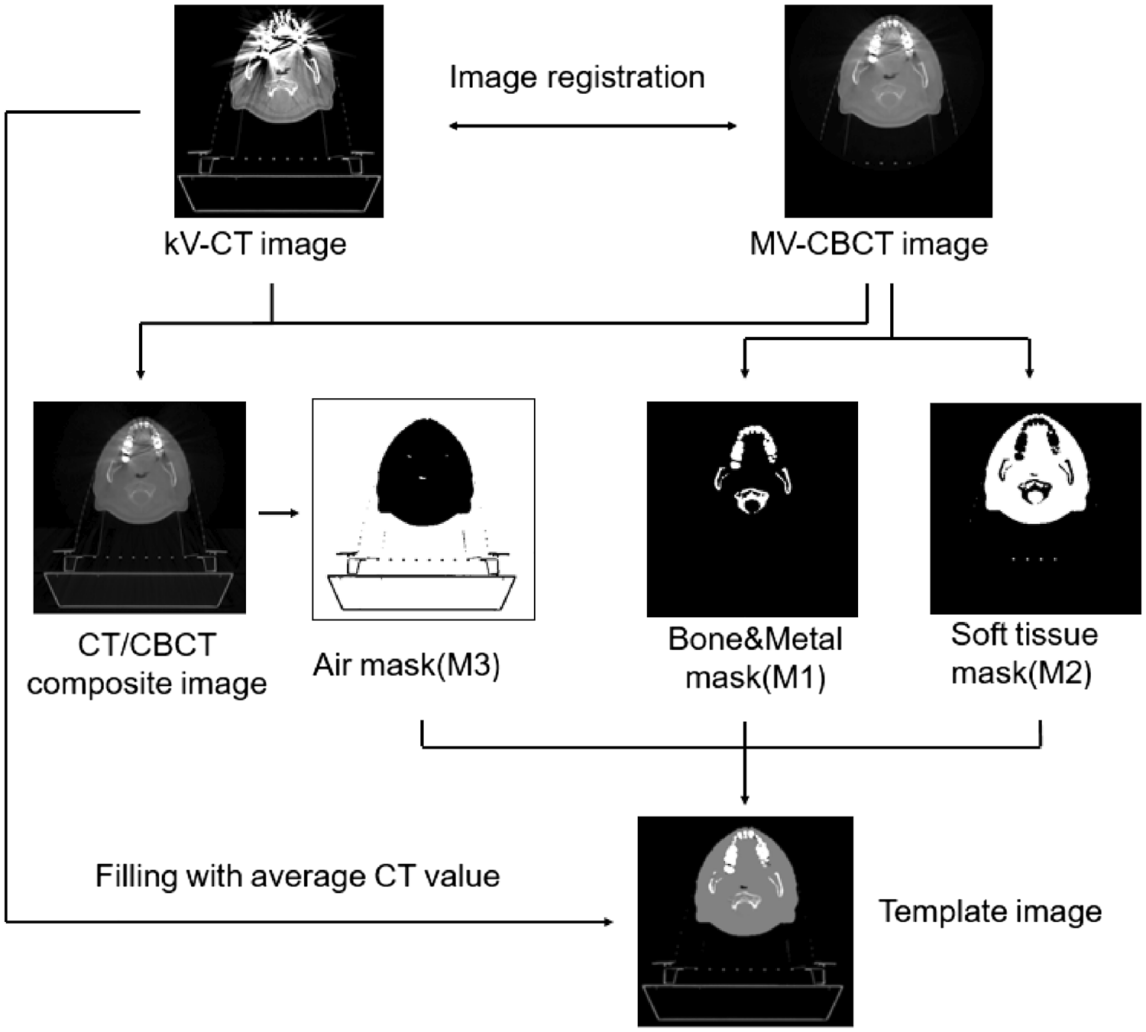
Template images generation process.

After the MV-CBCT images are registered with the kV-CT images, a pixel matrix of the same size as the kV-CT images are generated. In this step, the accuracy of metal artifacts reduction method will be depended on the quality of image registration between kV-CT and MV-CBCT, so all images (including artifact images and artifact-free images, but artifact images have lower weighting, the weighting value can be set to zero) are used in registration process to reduce the impact of artifact. For multi-metal images, there are still some metal artifacts in MV-CBCT images, the distribution of CT values caused by artifacts makes it difficult for global threshold segmentation to accurately extract different tissue masks. So the MV-CBCT images are divided into 8 * 8-pixel blocks in each artifact image, then we use the multi-threshold OTSU^30,31^ method (Maximum Inter-Class Variance Method) to segment each pixel blocks, and uses the OTSU method to determine the threshold between the high-density area M1 (including bone tissue and implanted metal area) and the medium-density area M2 (soft tissue area), and the threshold between the medium-density area M2 and the low-density area M3 (body cavity and extracorporeal air area). The segmentation results of different pixel blocks are spliced to obtain a complete tissue mask. Since the CT values of different tissues in the MV-CBCT images are quite different from the kV-CT images, the different regions of template images are filled with the CT values of the kV-CT images. The kV-CT images is first used as the template images *I*_*template*_ and then fill the different areas, as shown in formula (1):

1 $$ I_{template} = \& I_{template} \left( {i, \, j} \right) = \left\{ {\begin{array}{*{20}l} {I_{CT} \left( {i,j} \right),} \hfill & {\left( {i,j} \right) \in M1} \hfill \\ {\frac{{\mathop \sum \nolimits_{{\left( {i,j} \right) \in M2 }} I_{CT} \left( {i,j} \right)}}{{ N_{M2 } }},} \hfill & {\left( {i,j} \right) \in M2} \hfill \\ {\frac{{\mathop \sum \nolimits_{{\left( {i,j} \right) \in M3 }} I_{CT} \left( {i,j} \right)}}{{ N_{M3 } }},} \hfill & {\left( {i,j} \right) \in M3} \hfill \\ \end{array} } \right. $$

where* I*_*CT*_ is the kV-CT images matrix, *N*_*M2*_ and *N*_*M3*_ are the number of pixels in the areas M2 and M3 respectively. The entire processing flow is shown in Fig. 2.

### Images correction process

**Figure 3:**
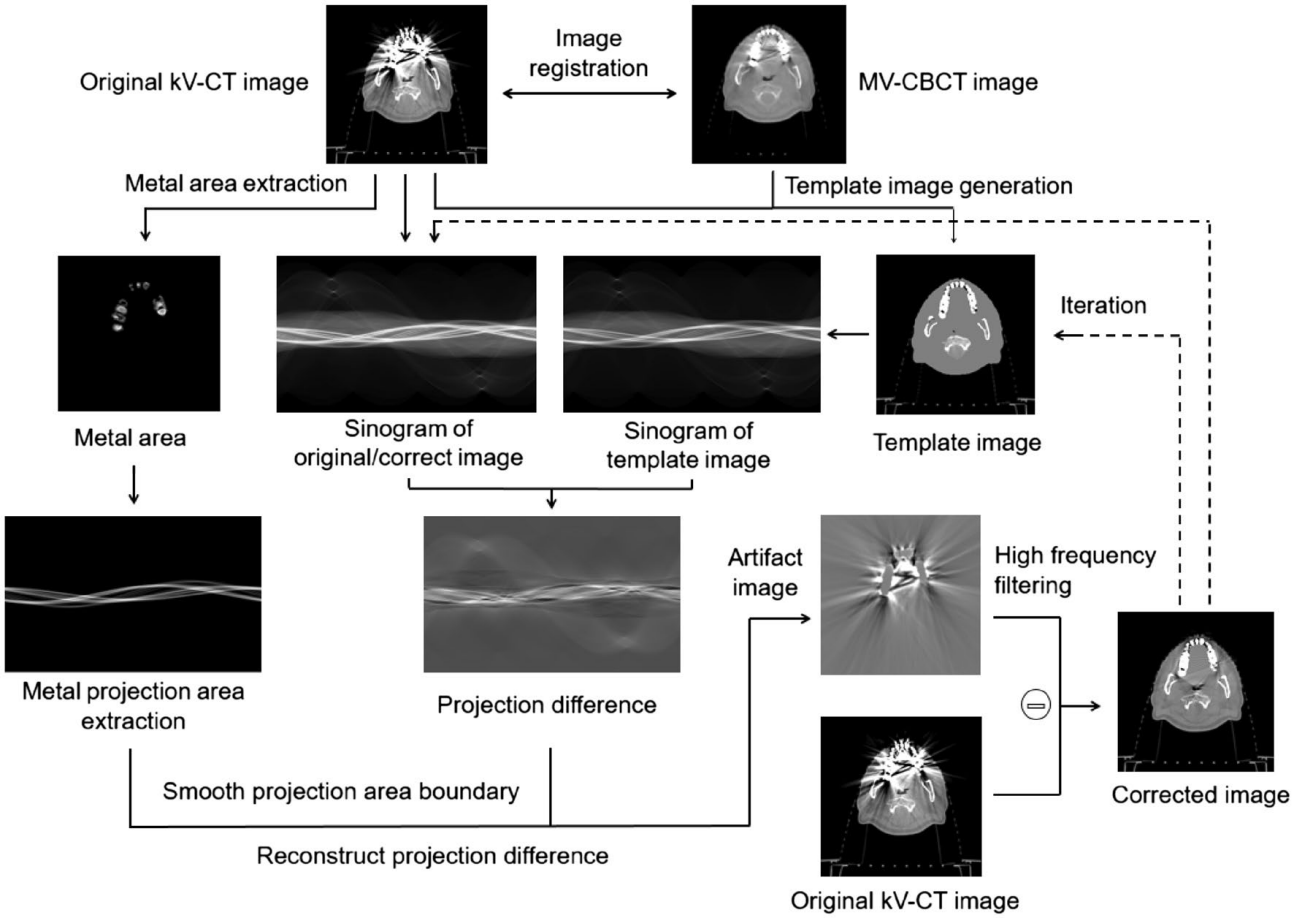
Flow chart of metal artifacts correction of proposed method.

After the template images are generated, the metal region *M*_*metal*_ is extracted by threshold segmentation in the kV-CT images, and the metal region is forward projected to generate a metal sinogram, and the non-zero region is the metal projection region. At the same time, the original kV-CT images *I*_*CT*_ and the template images *I*_*template*_ is forward projected to generate a sinogram. Since the CT values of different regions in the template images are filled with the average CT values of the corresponding regions in the kV-CT images, the difference in sinogram with kV-CT images mainly comes from the metal artifacts, and the formation of the metal artifacts is caused by the signal deviation on the beam path passing through the metal, so as long as the sinogram difference of the metal projection area is extracted, the kV-CT images can be corrected. The difference *P*_*MetalDiff*_ of the projection images of the metal area can be obtained from the above sinogram, as shown in formula (2):

2 $$ P_{MetalDiff} (\theta ,k) = FP(I_{CT} ) \, - FP(I_{template} ) \, ,(\theta ,k) \in FP(M_{metal} ) \ne 0 $$

where *FP* is the forward projection operator, *θ* is the projection angle, and *k* is the pixel position of the detector. After obtaining the sinogram difference *P*_*MetalDiff*_ of the metal projection area, the projection value is truncated in the boundary area, and new strip artifacts will be introduced in the subsequent processing. Therefore, the boundary area is smoothed so that the boundary projection value can be slowly reduced to zero, the linear interpolation processing method is used for smoothing, as shown in formula (3):

3 $$ P_{interp} (\theta , \, k) = \left\{ {\begin{array}{*{20}l} {P_{MetalDiff} \left( {\theta , k_{n1} } \right) - \frac{{P_{MetalDiff} \left( {\theta , k_{n1} } \right)}}{{\Delta_{n1} }}\left( {k - k_{n1} } \right),} \hfill & {k \in \left[ {k_{n1} - \Delta_{n1} ,k_{n1} } \right]} \hfill \\ {P_{MetalDiff} \left( {\theta , k_{n2} } \right) - \frac{{P_{MetalDiff} \left( {\theta , k_{n1} } \right)}}{{\Delta_{n2} }}\left( {k - k_{n2} } \right), } \hfill & {k \in \left[ {k_{n2} ,k_{n2} + \Delta_{n2} } \right]} \hfill \\ {P_{MetalDiff} \left( {\theta , k} \right),} \hfill & {k \in others} \hfill \\ \end{array} } \right. $$

where *k*_*n1*_ and *k*_*n2*_ are the upper boundary and lower boundary of a certain metal area under a certain projection angle, ${\Delta }_{n1}$ and ${\Delta }_{n2}$ is upper and lower boundary extension distance. The interpolated sinogram difference *P*_*interp*_ is back-projected to reconstruct the metal artifact images, and the artifact images is subjected to high-frequency filtering to reduce the high-frequency noise generated by the artifacts boundary and registration error. Artifact images is subtracted from kV-CT images to obtained corrected images, and the process can be expressed as:

4 $$ I_{correct} = I_{CT} {-} \, HFF\left[ {BP\left( {P_{interp} } \right)} \right] $$

where *BP* is the back-projection reconstruction operator, and *HFF* is the high-frequency filtering process. The above steps realize a complete correction process, but in this process, the CT value of the template images contains the influence of metal artifacts, which will cause the CT value of the corrected images in the artifacts area to be inaccurate. In the process of frequency filtering, the artifacts details will also be lost, and there will be some artifacts in the corrected images. Therefore, the template images ${I}_{template}^{iter}$ is generated again from the corrected images and brought into the correction process for iterative correction. The entire correction process is as follows Fig. 3.

### Evaluation strategy

In order to objectively and fairly evaluate the effects of various artifact correction methods, the CT value, noise, mean relative error (MRE), normalized root mean square deviation (NRMSD) and mean absolute deviation (MAD) of each corrected image and the reference image are analyzed statistically. In this study, MRE is used to measure the accuracy of CT value of the corrected image, and the standard deviation (SD) of pixel value in the range of interest (ROI) is used to measure the noise of CT image. MRE, NRMSD, and MAD are used to measure the relative and absolute difference between the specified image and the reference image, which can objectively evaluate the quality of CT images and are commonly used image evaluation indicators^27,32^. MRE, NRMSD, and MAD can be calculated by the formula (5)–(7):

5 $$ MRE = |I_{cor} {-} I_{ref} |/ |I_{ref} | $$

6 $$\mathrm{NRMSD}=\sqrt{\sum_{i\in ROI}{({I}_{i}^{cor}-{I}_{i}^{ref})}^{2}/\sum_{i\in ROI}{({I}_{i}^{ref})}^{2}}$$

7 $$MAD=\sum_{i\in ROI}|{I}_{i}^{cor}-{I}_{i}^{ref}|/N$$

where ${I}_{cor}$ is the mean CT value of corrected image in ROI and ${I}_{ref}$ is that of reference image, ${I}_{i}^{cor}$ is the CT value of corrected image in ROI and ${I}_{i}^{ref}$ is that of reference image. The smaller the value is, the closer the image is to the reference image and the higher the image quality is. The error bar is used to show the uncertainty of the statistical data.

After analyzing and processing the images data in this study, two senior doctors identified and scored the clarity of the anatomical structure, the degree of artifact suppression and the overall image quality in the image layer affected by metal artifacts. The Identifiability Score is 1 score: the structure cannot be identified, there are large artifacts; 2 scores: the structure can be recognized but no clear judgment can be made, the artifacts are obvious; 3 scores: acceptable, there are artifacts but still can observation; 4 scores: clear structure with only a few artifacts; 5 scores: very clear structure and no artifacts^32–34^.

### Statistical analysis

Statistical software IBM SPSS version 26.0 (IBM, Armonk, NY, USA) is used for statistical comparative analysis of scoring data. The statistical method is paired t-test, where *P* < 0.05 is considered statistically significant.

### Ethics approval and consent to participate

The experimental protocol was established, according to the ethical guidelines of the Helsinki Declaration and was approved by the Human Ethics Committee Eye & ENT Hospital, Fudan University. Written informed consent was obtained from individual or guardian participants.

## Results

### Images correction results and evaluation of CIRS phantom

**Figure 4:**
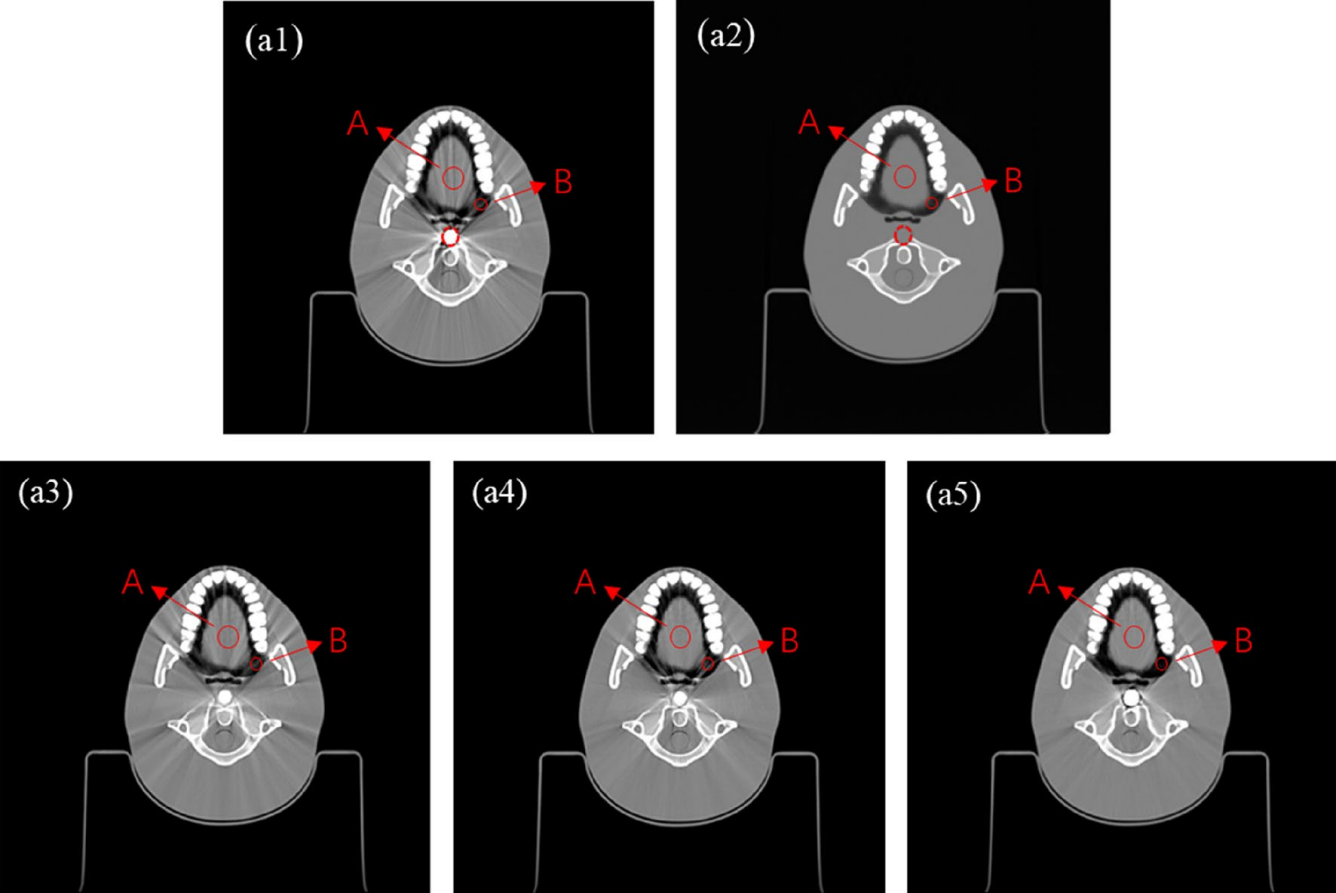
The origin and results of images corrected by different methods: (**a1**) Original kV-CT Image; (**a2**) Reference image without metal; (**a3**) LIMAR method; (**a4**) NMAR method; (**a5**) Proposed method.

Firstly, the simulation head phantom CIRS model 038 is used to verify the correction method. A metal rod is artificially placed in the simulation phantom, which causes obvious radial metal artifacts, as shown in Fig. 4(a1). The CT images of the CIRS phantom without a metal rod are not contaminated by metal artifacts, so it is used as reference images, as shown in Fig. 4(a2). Figure 4(a3)–(a5) is the corrected image of the LIMAR^3^, and NMAR^4^ methods and the proposed method, as shown in the corrected image, some metal artifacts are removed after applying the correction method, and the artifact removal effect of the proposed method is more significant.

As shown in Table 1, CT value, noise, MRE, NRMSD and MAD of different methods are compared. Reference values are calculated in reference image without metal. In the image corrected by the proposed method, the CT value is 49.6HU, the noise is 18.2HU, MRE is 0.03, NRMSD is 15.21, and the MAD is 0.40 in region A. Among all methods, the calculated values of proposed method is closest to reference values, it can achieve the best image performance index.

**Table 1:** The average CT value, noise, MRE, NRMSD and MAD in different regions of original images and correction images of different methods.

	Original images	LIMAR	NMAR	Proposed method	Reference
Region A					
CT Value (HU)	28.4	12.9	39.1	**49.6**	**48.0**
Noise (HU)	58.7	30.9	26.4	**18.2**	**9.1**
MRE	0.41	0.73	0.19	**0.03**	**0**
NRMSD	38.5	29.28	20.86	**15.21**	**0**
MAD	1.09	0.78	0.54	**0.40**	**0**
Region B					
CT value (HU)	− 501.5	− 512.6	− 518.3	**− 678.4**	**− 698.4**
Noise (HU)	91.3	73.6	150.2	**33.8**	**31.1**
MRE	0.28	0.27	0.26	**0.03**	**0**
NRMSD	166.95	138.77	158.8	**35.22**	**0**
MAD	0.24	0.22	0.24	**0.05**	**0**

Significant values are in bold.

**Figure 5:**
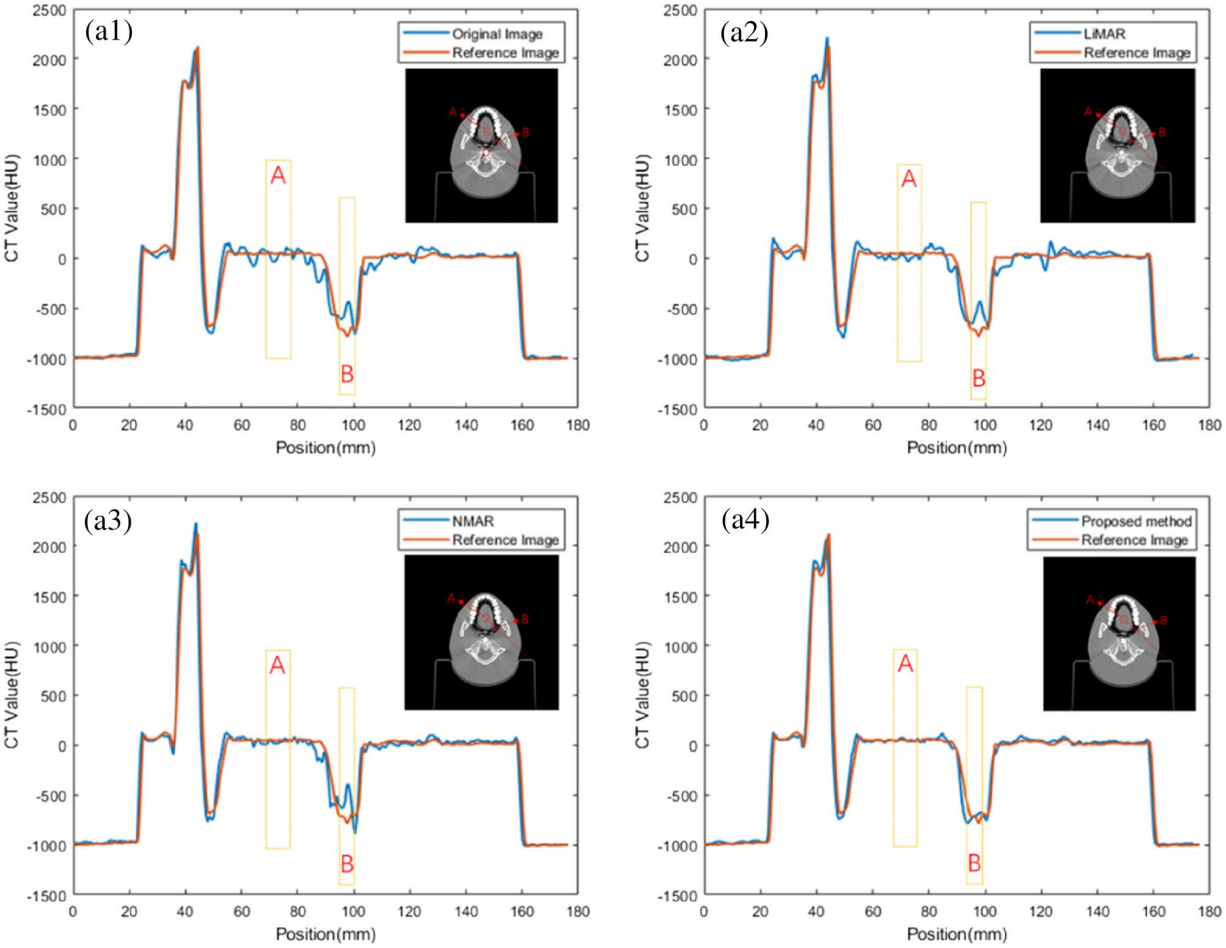
Profiles of CT values at the line (red line) pass through the region A and B: (**a1**) Original image; (**a2**) LIMAR corrected image; (**a3**) NMAR corrected image; (**a4**) Proposed method corrected image.

The noise suppression effect of proposed method is particularly prominent. Figure 5 shows the profiles of CT values at the line passing through the region A and B, it can be found that the fluctuation amplitude of the profile of the proposed method is the smallest of all methods, which proves the superior noise suppression performance.

### Images correction results and evaluation of clinical patients

**Figure 6:**
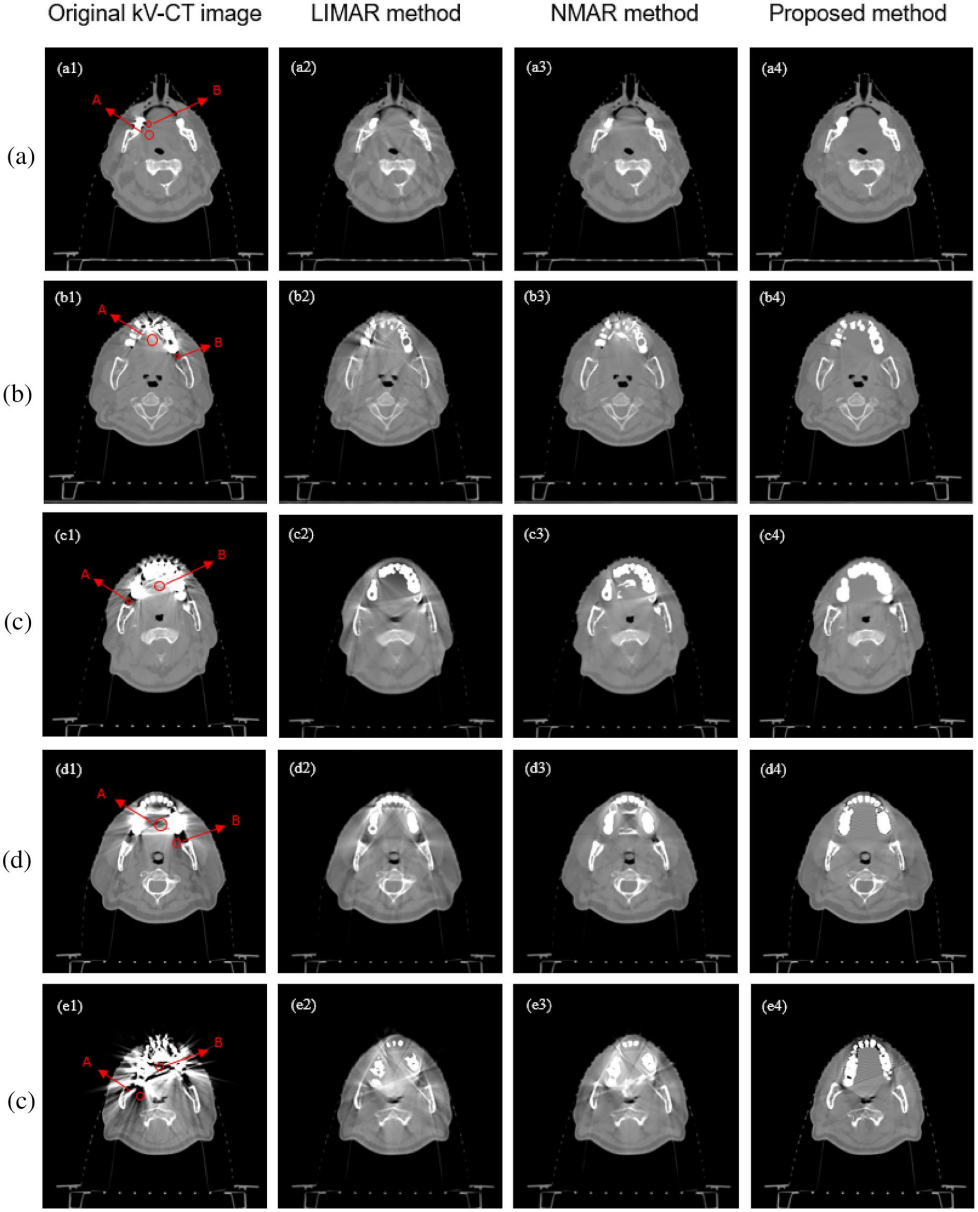
Correction result of different methods: (**a**–**e**) 5 selected image groups.

At the same time, the correction method is applied to the kV-CT images of real radiotherapy cases. The registration accuracy in proposed method is about 0.5 mm in each direction base on artifact-free image slice. CT data set of 7 patients are used in this study, 5 slice images of different patients are selected to show correction result, as shown in Fig. 6. Because the patient has implanted multiple metal dentures, the kV-CT images have very serious and mutually influencing metal artifacts. Use the method in this paper to process the data, and use LIMAR and NMAR methods to process the same layer of data as reference comparison images, the processing results are shown in Fig. 6, the following images have been adjusted to the same window width and window level.

As shown in Fig. 6 (A/B area in red circle), the same areas in different groups of images are selected, to calculate the average CT value and the variance of CT values in the area which can comparing CT value accuracy and noise of different correction methods. A and B are the lighter and darker areas in the same issue.

CT image performance indicators such as CT value, noise and MRE in ROI are compared to quantitatively evaluate the correction results of these methods. For patient CT data, the images are contaminated and there is no theoretical reference image. Considering that the ROI selected is small enough, the internal tissue is uniform, and the middle value of the CT value range of the corresponding anatomical tissue of ROI is used as the reference value.

As shown in Table 2, due to the influence of metal artifacts, the reference values cannot be accurately obtained. Therefore, the CT values of the surrounding artifacts-free areas are used instead, and a rough range of CT values is given. Different correction methods can improve the accuracy of images CT value and reduce the inhomogeneity of CT value to a certain extent. Through the comparison of statistical results, the method used in this paper can achieve better correction result than other correction methods, and the CT value of the corrected images is closer to the reference CT value.

**Table 2:** The average CT value and noise of 5 image groups in different regions.

	CT mean value and noise (HU)	Original kV-CT images	LIMAR	NMAR	Proposed method	Reference CT value
(a)	Region A	2.4 ± 28.7	58.6 ± 34.7	35.8 ± 21.7	**6.7 ± 12.6**	− 30 to 60
	Region B	− 71.4 ± 49.3	18.2 ± 55.8	53.7 ± 70.2	**− 5.6 ± 7.4**	− 30 to 60
(b)	Region A	358.6 ± 110.3	169.3 ± 72.4	391.3 ± 254.7	**17.8 ± 16.3**	− 94 to 40
	Region B	− 480.2 ± 176.2	− 106.4 ± 45.7	− 175.8 ± 73.5	**− 38.1 ± 14.6**	− 94 to 40
(c)	Region A	338.3 ± 92.8	− 77.7 ± 37.9	147.1 ± 19.1	**48.6 ± 13.4**	− 60 to 70
	Region B	− 601.4 ± 146.2	− 99.2 ± 31.5	− 79.8 ± 86.6	**− 65.3 ± 54.0**	− 60 to 70
(d)	Region A	113.6 ± 303.9	32.0 ± 41.6	45.7 ± 167.1	**54.8 ± 33.8**	− 80 to 50
	Region B	− 289.5 ± 108.4	− 66.6 ± 73.5	38.5 ± 36.9	**33.1 ± 50.8**	− 80 to 50
(e)	Region A	− 389.6 ± 813.2	247.4 ± 99.2	222.1 ± 95.6	**31.0 ± 61.0**	− 100 to 54
	Region B	− 947.1 ± 133.4	45.4 ± 149.5	− 72.6 ± 102.4	**− 64.6 ± 61.3**	− 100 to 54

Significant values are in bold.

**Figure 7:**
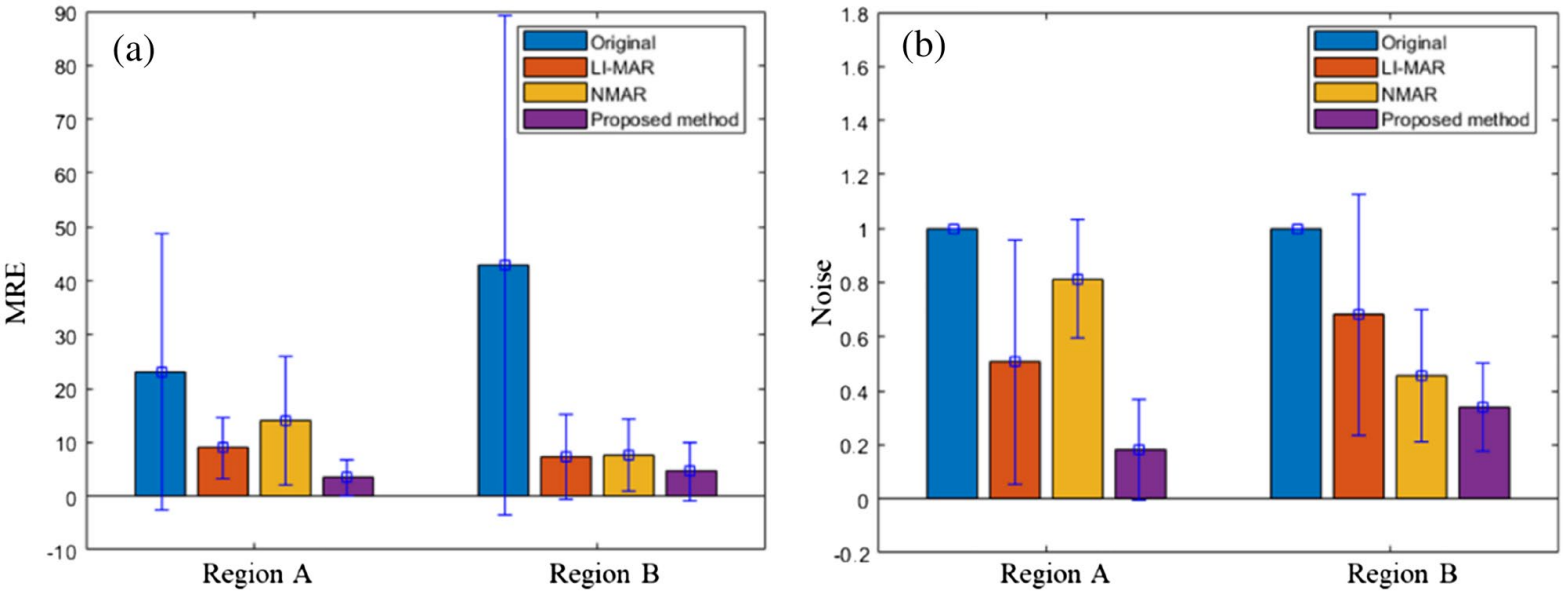
Comparison of the statistical results of different groups of data corrected by different methods: (**a**) MRE; (**b**) Image noise normalized with noise value of original CT images.

MRE and noise suppression effects of different methods are compared. Figure 7 shows that the average MRE of the proposed method can reach 4.1, and the noise can be reduced to 26.1% of the original image. Compared with LIMAR, the MRE is reduced by 50.5%, and the noise is reduced by 56.2%; Compared with the NMAR method, MRE is reduced by 63.3% and noise is reduced by 58.9%. Among all methods, this method can achieve the better image performance index.

### Identification and evaluation of images anatomy

The degree of artifacts suppression and the overall images quality in the images have been identified and scored the clarity of the anatomical structure, the degree of artifacts suppression and the overall images quality in the images layer affected by metal artifacts by senior doctors. The bone window setting (window level 450, window width 1600) is used to identify and score high-density bony structures such as teeth and upper/mandibular bones. The soft tissue window setting (window level 40, window width 400) is used for identify and score medium-density structures such as the tongue, lip and muscle. The lung window setting (window level -400, window width 1500) is used to identify and score low-density cavities in the oral cavity and pharynx. As shown in Table 3, the Score of corrected images by proposed method are higher than original images, and the differences between original images and corrected images in different body parts are all significant (*P* < 0.05), which indicates that proposed method can greatly improve image quality.

**Table 3:** Identifiability Score for original images and corrected images in this study.

Body part	Scoring for original images	Scoring for corrected images in this study	*P* value
Tooth	2.43 ± 0.79	4.00 ± 0.58	0.000
Upper/lower jaw	2.57 ± 1.40	4.14 ± 0.47	0.008
Tongue	2.86 ± 1.21	3.86 ± 0.90	0.004
Lips	2.57 ± 0.79	3.86 ± 0.38	0.000
Masseter muscle	2.57 ± 1.13	4.43 ± 0.79	0.000
Cavity	2.86 ± 0.90	4.86 ± 0.37	0.001

## Discussion

The LIMAR obtains the metal area projection by linearly interpolating the two-sided projection values of the metal area projection. This method reduces the artifacts brought by the back-projection reconstruction of the metal area to a certain extent, but the interpolation area will cause the loss of projection information. A better correction can be achieved for the artifacts caused by a single metal implant with a small volume. However, in the case of multiple metals or large metal implants, the use of linear interpolation will cause too much loss of projection information, and new artifacts will be introduced or some details will be lost in the reconstructed images.

The method based on a prior images NMAR uses the original images to obtain the prior images, normalizes the projection value of the original images through the projection value of the prior images, and then interpolates the metal area. The discontinuity of metal region interpolation is better than LIMAR in image repair, but when the metal artifacts are serious, it is difficult to obtain ideal prior images, and the image correction result is limited.

The image data used in this paper were obtained on the CT-accelerator-integrated uRT-linac 506c linear accelerator system produced by Shanghai United Imaging Healthcare Co., Ltd. The uRT-linac 506c accelerator contains an MV-CBCT imaging system and a diagnostic kV-CT imaging system. The original design concept of this type of accelerator integrates diagnostic CT and linear accelerator into one treatment room. The patient can complete the image scanning of MV-CBCT and kV-CT at one time in the same treatment room and the same scanning position without leaving the couch, and the obtained images can be perfectly fused and registered. It avoids the inevitable deformation error during image registration after the patient scans images with different equipment.

The patients enrolled in this study all have multiple metal-implanted dentures that cannot be removed before scanning. Conventional kV-CT localization images will show severe artifacts, and the tissue structure cannot be identified, metal artifacts seriously affect target identification and organ-at-risk delineation^2^. After the images data were analyzed and processed by the method of this study, it was identified and scored by more than 2 senior doctors. Compared with the original reference images, under the bone window setting (window level 450, window width 1600), the recognition of bony structures in the processed images has the most improvement, including: teeth, maxillary/mandibular, and other bone structures can clearly distinguish the boundary between bone and surrounding soft tissue, but there are still subtle artifacts. Under the soft tissue window setting (window level 40, window width 400), the structure of the soft tissue affected by the artifacts is greatly improved, and the soft tissue such as the front lip and the masseter muscle on both sides can be clearly distinguished, but there are still small artifacts in the adjacent part of the tongue and metal implantation. Since the density of the cavity in the oral cavity and the pharyngeal cavity is uniform, and the density of the surrounding bone tissue and soft tissue is quite different, the lung window setting (window level -400, window width 1500) is used to identify and evaluate them, cavity contours are clear in the processed images and most of the effects of metallic artifacts have been eliminated.

This study also has certain limitations. The patient needs to scan CBCT once more and receive the dose of CBCT once more, and the image process time is increased under the same hardware conditions. In addition, this method can be extended to other sites body parts in theory, but due to the lack of clinical data on other body parasites, no further research has been carried out.

## Conclusion

At present, several existing correction algorithms for metal artifacts in CT images have certain effects, but there are still many limitations in practical applications. In this paper, a unique artifacts correction method is proposed. Unlike the other methods, the MV-CBCT images are used to obtain additional tissue structure information, and the iterative method is used to obtain accurate CT values and gradually repair the details, a better correction can be achieved especially for the complex artifact images caused by multi-metal interaction. The average MRE of the proposed method can reach 4.1, and the noise can be reduced to 26.1% of the original image. Compared with LIMAR, the MRE is reduced by 50.5%, and the noise is reduced by 56.2%; Compared with the NMAR method, MRE is reduced by 63.3% and noise is reduced by 58.9%. The Identifiability Score of the tooth, upper/lower jaw, tongue, lips, masseter muscle and cavity in the corrected images by the proposed method have significantly improved (*P* < 0.05) than original images. The combination of MV-CBCT images and kV-CT images of the same body position can effectively remove metal artifacts in CT images, improve the resolution of various tissues and organs in the images, and greatly improve the images quality of kV-CT images, especially for polymetallic and complex metal implants. It can also achieve a better correction result compared with other methods, which can provide a solid foundation for delineating target areas and dose distribution calculation.

## Data Availability

The datasets used and analyzed during the current study are available from the corresponding author upon reasonable request.

## Abbreviations

CT: Computed tomography
CBCT: Cone beam computed tomography
M1: Including bone tissue and implanted metal area
M2: Soft tissue area
M3: Body cavity and extracorporeal air area
LIMAR: The linear interpolation
NMAR: The normalized interpolation
